# Distinct Responses of Corrosion Behavior to the Intermetallic/Impurity Redistribution During Hot Processing in Micro-Alloyed Mg Alloys

**DOI:** 10.3390/ma18235473

**Published:** 2025-12-04

**Authors:** Yiming Jin, Hong Yang, Jan Bohlen, Björn Wiese, Yan Su

**Affiliations:** 1 Southwest Technology and Engineering Research Institute, Chongqing 401329, China; suyan71@126.com; 2 Institute of Metallic Biomaterials, Helmholtz-Zentrum Hereon, 21502 Geesthacht, Germany; bjoern.wiese@hereon.de; 3 National Engineering Research Center for Magnesium Alloys, Chongqing University, Chongqing 400044, China; hong.yang@cqu.edu.cn; 4 College of Materials Science and Engineering, Chongqing University, Chongqing 400044, China; 5 Institute of Material and Process Design, Helmholtz-Zentrum Hereon, 21502 Geesthacht, Germany; jan.bohlen@hereon.de

**Keywords:** magnesium, alloy, corrosion, intermetallics, impurities, hot processing

## Abstract

By tuning the extrusion parameters, the corrosion performances of as-extruded Mg-0.5Zn(-0.2X) alloys (X: Ca/Sr/Ag/In/Cu, denoted as Z05, Z0502-Ca, Z0502-Sr, Z0502-Ag, Z0502-In and Z0502-Cu, respectively) with similar grain sizes were investigated and compared with their as-cast counterparts. The formed Fe-Si precipitates after hot processing significantly accelerate the corrosion rates of Z05, Z0502-Ag and Z0502-In, whereas the driving force from the Fe-encapsulated MgCaSi(Fe) and MgSrSi(Fe) precipitates are not as strong in Z0502-Ca and Z0502-Sr. Impacts from Fe impurity in Z0502-Cu are masked in the fast corrosion due to the noble Mg2Cu intermetallics. Fe precipitation during hot processing is critical for micro-alloyed systems, as the changes in intermetallic/impurity distributions impact the corrosion performances profoundly. The enthalpy of formation and the potential difference are the key factors that influence the distribution of precipitate during hot processing.

## 1. Introduction

Mg is one of the lightest metals and Mg alloys have promising applications in fields requiring light weight due to their high strength-to-weight ratio. Recently, micro-alloyed Mg systems have attracted much attention as the dilute additions of alloying elements can induce remarkable influences on the performances [1,2] while not forming abundant intermetallics [3]. Due to the inadequate intrinsic properties of as-cast micro-alloyed Mg products [4], the combination of heat treatment and thermomechanical processing proves to be a useful approach [5,6]. Dissolution of the intermetallics derived from solution annealing [7,8] and fine grains derived from extrusion/rolling [9,10] lead to improved corrosion performances. Apart from the processing routes, another key factor that influences the corrosion performance is the inherent impurities. These impurities are usually nobler regarding the electrochemical potential and can effectively drive the electrochemical reactions [11]. The tolerance limits for Fe, Ni and Cu impurity in pure Mg are 180 ppm, 5 ppm and 1000 ppm, respectively, and once the threshold is exceeded, the corrosion rate of Mg will increase by orders of magnitude [12,13]. Among these, Fe is one of the most common impurities.

Although the Fe tolerance limit of as-cast pure Mg is 180 ppm according to the Mg-Fe phase diagram, that of heat-treated pure Mg reduced distinctly to less than 10 ppm [14]. Similarly, accelerated corrosion rates of Mg-0.3Ca, Mg-0.3Si, Mg-0.1Sr and Mg-6Al after solution heat treatment [15] and Mg-X alloys (X = 5Gd, 0.3Ca, 6Al, 1Mn, 5Sn, 0.1Sr, 0.6Nd, 0.7La, 0.9Ce) after annealing and hot rolling [16] were reported, as there might be higher sensitivities to the precipitated harmful particles during hot processing, whereas no direct evidence was shown. One might argue that multiple studies have already shown the significant downscale of corrosion kinetics after hot processing [8,17]. However, the complex consequences might still be related to the amount, solubility and interaction potency of the alloying elements and impurities. Recently, Jin et al. [18] unveiled the preferred formation of Fe-Si precipitates in Mg-Zn alloy, and their corresponding Fe concentrations increased as the annealing duration extended, which activated the corrosion initiation and propagation vigorously. Moreover, solution annealing seemed to poison the effects of extrusion, as the annealed and then extruded sample corroded much faster than the cast and then directly extruded sample.

To date, attempts like adding Zr in Mg melts [19], low temperature melt treatment [20] and inorganic [21] and organic acid pickling [22,23] have been made to address the impurity-induced corrosion problem. However, these methods either deal with the melt purification or surface contamination removal. Interactions between intermetallics and impurities are important, yet the related efforts are still quite limited. Al_8_Mn_5_ was widely recognized to capture Fe to realize better corrosion resistance [14,24], whereas the isolated Fe-Si particle could accelerate the corrosion rate [18]. Moreover, for the micro-alloyed Mg alloys, the diminishment of intermetallics accompanies the redistribution of harmful particles during annealing, leaving the response of corrosion behavior even more diversified. Hence, to understand the interactions between intermetallics and impurities during hot processing, and to add more information to the existing processing impurity-related database that is mostly based on pure Mg [25,26,27] and Mg-Al alloys [28,29], the corrosion behaviors of the as-extruded Mg-0.5Zn(-0.2X) alloys are systematically compared to evaluate their sensitivities to hot processing in this study. To fabricate systems with different intermetallic compounds, elements with different solubility in Mg are considered. Moreover, due to the potential bio-application of micro-alloyed Mg alloys, the elements should also have good biocompatibility. Thusly, the X element is selected as Ca, Sr, Ag, In and Cu, and the corresponding alloys are denoted as Z05, Z0502-Ca, Z0502-Sr, Z0502-Ag, Z0502-In and Z0502-Cu, respectively. The biocompatiblity and solubility of the selected alloying elements are shown in Table 1. The grain sizes of the as-extruded (hereafter shortened as EX) alloys are tuned so that the effects of grain size can be neglected. As a continuous work of ref. [30], the corrosion behavior of the as-cast and EX Mg-0.5Zn(-0.2X) alloys are also compared. The experimental data and revealed mechanisms can provide guidance for the subsequent forward design of micro-alloyed Mg systems.

## 2. Materials and Methods

### 2.1. Material Preparations

**Figure 1:**
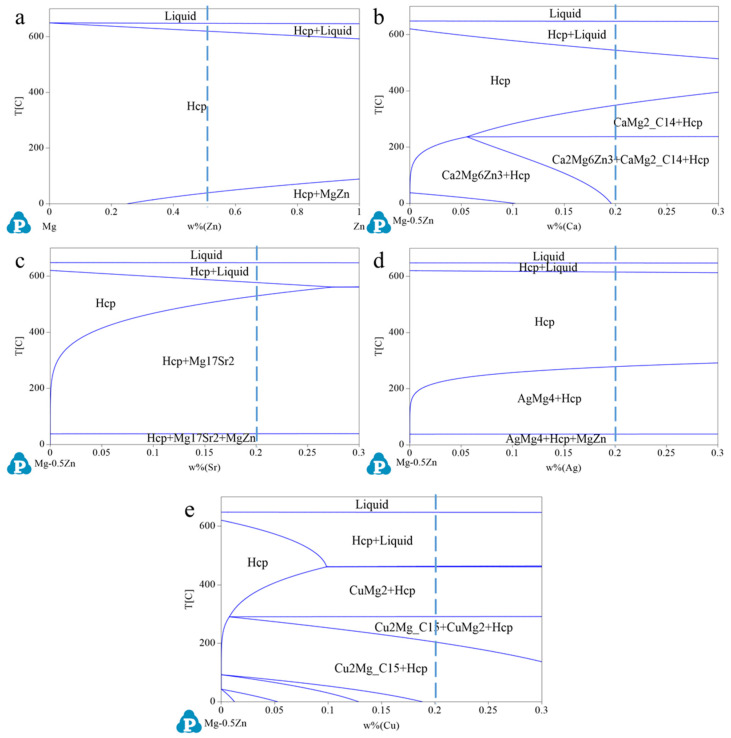
Thermodynamic-calculated phase diagrams of (**a**) Z05, (**b**) Z0502Ca, (**c**) Z0502Sr, (**d**) Z0502Ag and (**e**) Z0502Cu systems by Pandat^TM^ 2017 with PanMagnesium 2017 database. The blue dash line represents the exact composition of each system.

The as-cast Mg-0.5Zn(-0.2X) alloys were prepared following the procedures in our previous study [30]. The casting ingots were machined into cylindrical billets (150 mm length × 50 mm diameter) for further use. To homogenize the microstructures, solution annealing was performed. The heat treatment parameters for Z05, Z0502-Ca, Z0502-Sr, Z0502-Ag and Z0502-Cu systems were derived from the phase diagrams (Figure 1) calculated by Pandat software (PanMagnesium 2017 database [31], CompuTherm LLC, Middleton, WI, USA) and that for Z0502-In was from Mg-In binary phase diagram [32]. Z05, Z0502-Ag, Z0502-In and Z0502-Cu alloys share the same annealing parameters (400 °C for 16 h). Z0502-Ca alloy was treated at 450 °C for 16 h while Z0502-Sr was treated at 530 °C for 8 h under Ar protective gas due to safety considerations. All the annealed ingots were water-quenched at ambient temperature and then pre-heated at 350 °C for 1 h. The dyes and press blocks were also pre-heated at 350 °C to avoid temperature gradient. Following that, indirect extrusion was conducted at 350 °C with multiple extrusion speeds (0.6, 2.2 and 4.4 mm/s). The extrusion ratio ϕ was 25:1, which can be calculated as ϕ = A_0_/A_f_, where A_0_ and A_f_ are the transverse area of the container and the profile, respectively. The extruded bars were air-cooled at room temperature. The diameter and length of the extruded bar were 10 mm and around 320 cm, respectively. Compositions of the samples were measured via Inductively Coupled Plasma-Optical Emission Spectroscopy (ICP-OES, Thermo Fisher Scientific iCAP duo 6500, Waltham, MA, USA) and the results are shown in Table 2.

### 2.2. Microstructure Characterization

**Figure 2:**
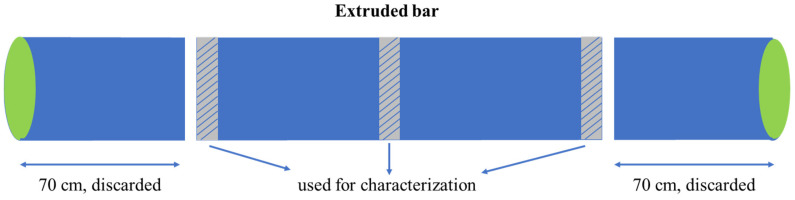
Schematic of the specimen used for characterization.

The first 70 cm and last 70 cm of the extrusion bar were discarded and the in-between part was used. During the extrusion process, only the middle part of the extrusion bar has a homogeneous microstructure, whereas a steady state is not guaranteed in the beginning and ending parts. To verify the homogeneity, specimens at the two ends and midpoint of the remaining bar were used for characterization (Figure 2).

The specimens were wet ground with SiC abrasive paper up to 2500 grit consecutively and polished with a mixture of water-free silica colloid (OPS^TM^), 1 µm diamond slurry and ethylene glycol/ethanol-based lubricant using an automatic polishing machine. The grain structures were revealed by optical microscopy (OM). The grain size was measured according to a line intercept method [33] using the software analySIS pro 5.0. The influences of extrusion speeds on the alloy grain sizes are shown in Table 3.

Scanning electron microscopy (SEM) equipped with energy dispersive spectroscopy (EDS) were utilized to characterize the microstructures and compositions. Texture measurements were carried out using a Malvern Panalytical X-ray diffractometer with goniometer setup and Cu Kα radiation. The pole figures of the first six peaks were measured using a beam size of 1.5 mm × 1 mm within a grit of 5° × 5° with full rotations and a maximum tilt angle of 70°. An open source code MTEX [34] was applied to recalculate the full orientation distribution function ODF from the measurements. Inverse pole figures in the extrusion direction (ED) as well as maximum pole density levels were used for evaluation, as the ED is the only single distinct direction within the sample geometry within its rotational symmetry. The Miedema model (Equations (1) and (2)) [35,36,37] was referred to for the acquisition of the standard molar enthalpies of formation (ΔH) in the i-j binary systems:

(1) $$∆H_{ij}=f_{ij}\frac{x_{i}(1+{µ}_{i}x_{j}\left(\emptyset_{i}-\emptyset_{j}\right))x_{j}(1+{µ}_{j}x_{i}\left(\emptyset_{j}-\emptyset_{i}\right))}{x_{i}V_{i}^{2/3}(1+{µ}_{i}x_{j}\left(\emptyset_{i}-\emptyset_{j}\right))+x_{j}V_{j}^{2/3}(1+{µ}_{j}x_{i}\left(\emptyset_{j}-\emptyset_{i}\right))}$$

(2) $$f_{ij}=\frac{2pV_{i}^{2/3}V_{j}^{2/3}(q/p{\left({\left(n_{ws}^{1/3}\right)}_{j}-{\left(n_{ws}^{1/3}\right)}_{i}\right)}^{2}-{\left(\emptyset_{i}-\emptyset_{j}\right)}^{2}-\alpha (\frac{r}{p}))}{{{\left(n_{ws}^{1/3}\right)}_{i}}^{-1}+{{\left(n_{ws}^{1/3}\right)}_{j}}^{-1}}$$

where x, V, Φ and n_ws_ represent the molar fraction, molar volume, electronegativity and electron density of alloying element i and j, respectively. The values of the empirical parameters μ, p, q, r and α are referred to from ref. [38].

### 2.3. Characterization of Corrosion Performance

The corrosion tests were conducted in 0.9 wt.% NaCl solution at room temperature. Potentiodynamic polarization (PDP) and electrochemical impedance spectroscopy (EIS) were conducted with a potentiostat connected to a three-electrode cell. The exposed area of the specimen and the volume of the electrolyte were 0.5 cm^2^ and 330 mL, respectively. The electrolyte was constantly agitated by a magnetic stirrer at 200 rpm. After 30 min open circuit potential (OCP) measurement, PDP tests commenced from −150 mV with respect to the free corrosion potential (scanning rate being 0.2 mV/s). By selecting the linear part of the cathodic branch (~50 mV with respect to the corrosion potential) via ACM Analysis software, the corrosion current density (i_corr_) was obtained from its interception with the vertical corrosion potential line. After stabilizing OCP for 5 min, EIS spectra were acquired under OCP after 0, 1, 2, 3, 6, 12, 24, 48 and 72 h, respectively. The frequency range was from 30,000 Hz to 0.1 Hz, and the sinusoidal perturbation was 10 mV. The EIS spectra were fitted using Zview software (ZView 2, Scribner Associates Inc., Southern Pines, NC, USA). A hydrogen evolution test was also performed. Since the outmost surface of the wrought material might be impurity-enriched [21,22], the extruded bar was further machined into rods (9 mm diameter × 20 mm length). The dimensions of all samples were recorded to calculate the exposed surface areas. To avoid contact with the container, the sample was hung in the medium with a nylon fishing wire. The container was sealed with Parafilm to keep the electrolyte volume at 350 mL. The corrosion rate derived from H_2_ volume P_H_ (mm/year) was calculated as $P_{H}=2.088\;V_{H}$, as referred to from ref. [39].

## 3. Results

### 3.1. Microstructure

**Figure 3:**
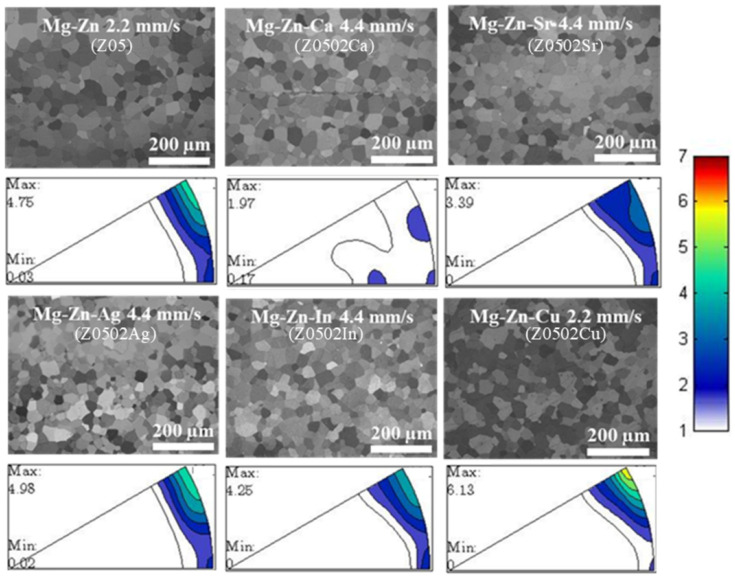
OM micrographs and IPFs of the EX Mg-0.5Zn(-0.2X) alloys. Lower left ⟨0001⟩ pole, lower right $\left\langle 11\bar{1}0\right\rangle$ pole and upper right $\left\langle 11\bar{2}0\right\rangle$ pole. Max and Min indicate the maximum and minimum pole density, respectively.

To exclude the influence of grain size [9,40,41,42], only the extruded Mg-0.5Zn(-0.2X) alloys with fully recrystallized grain structure and similar grain sizes are selected for further study, i.e., Z05 and Z0502-Cu extruded at 2.2 mm/s, Z0502-Ca, Z0502-Sr, Z0502-Ag and Z0502-In extruded at 4.4 mm/s. All these alloys have equiaxed grains, with the average grain size being around 30 μm (Figure 3). According to the IPFs, all the EX Mg-Zn(-X) alloys have typical fiber texture (c-axes⊥ED), apart from Z0502-Ca. The texture intensity of Z05 is greatly reduced from 4.75 to 1.97 by adding Ca, and a “rare earth” component can be observed, which is featured by an intensities tilt toward the 〈0001〉 pole between the $\left\langle 10\bar{1}0\right\rangle$ and $\left\langle 11\bar{2}0\right\rangle$ poles [43]. The addition of Sr also helps to reduce the texture intensity, whereas the addition of Cu seems to increase the texture intensity.

**Figure 4:**
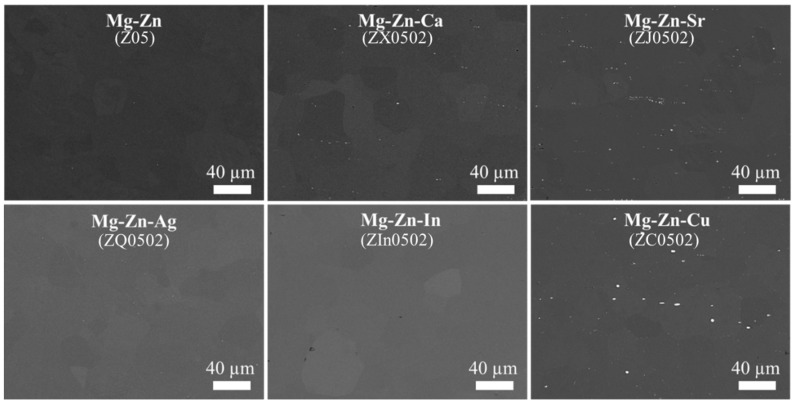
SEM Microstructures of the EX Mg-0.5Zn(-0.2X) systems.

**Figure 5:**
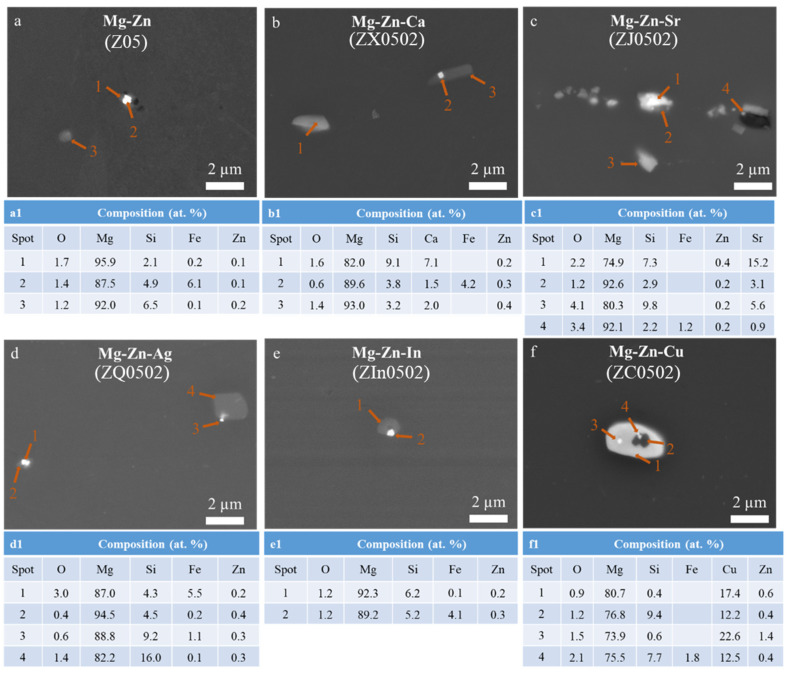
(**a**–**f**) SEM micrographs of the intermetallics in EX Mg-0.5Zn(-0.2X) and (**a1**–**f1**) the corresponding EDS results.

SEM microstructures along the longitudinal cross sections of the EX Mg-Zn(-X) alloys are revealed in Figure 4. The dendritic microstructures and the Zn segregations existing between the dendritic arms from the as-cast state [30] are dissolved after heat treatment and extrusion. Z0502-Cu, Z0502-Sr and Z0502-Ca show comparatively higher amounts of intermetallics, with the area fractions being 0.26, 0.23 and 0.10%, respectively, whereas the low amounts of intermetallics in Z05, Z0502-Ag and Z0502-In defy quantification. From the SEM images in higher magnification (Figure 5), the EX Z05, Z0502-Ag and Z0502-In samples have analogous coexisting intermetallic particles (CE-IMP) containing Fe/Si and Mg/Si precipitates. The Fe/Si precipitates are usually submicron-sized and the Mg/Si precipitates are bigger (1–2 µm in size). Some isolated Mg/Si precipitates can also be discerned. Mg/Ca/Si-containing intermetallics are found in Z0502-Ca. Particles with different dominant elements in concentration (Si < Sr, Si ≈ Sr, Si > Sr) exist in Z0502-Sr. These precipitates differ significantly in size and tend to distribute in parallel to the ED. Occasionally, Fe-incorporated Mg/Ca/Si and Mg/Sr/Si precipitates can also be recognized in these systems. Precipitates varying from 5 µm to hundreds of nm in size can be found in Z0502-Cu, which are enriched in Cu (10–20 at.%). CE-IMP containing Mg/Cu and Mg/Cu/Si precipitates exist in Z0502-Cu, while Fe tends to be incorporated in the Mg/Cu/Si part. It is generally found that the Zn-containing intermetallics dissolved after hot processing, whereas the Si-containing intermetallics can still be detected, indicating their relatively high thermostability.

### 3.2. Corrosion Performance

The corrosion performances of EX Mg-0.5Zn(-0.2X) alloys are characterized by different methods, which have their own unique benefits and limitations [44]. Different methods, in their response to local surface heterogeneity and film formation, may yield conflicting trends. Therefore, the authors employed electrochemical (PDP, EIS) and non-electrochemical methods (hydrogen evolution, corrosion morphology) aiming for a more convincing evaluation.

**Figure 6:**
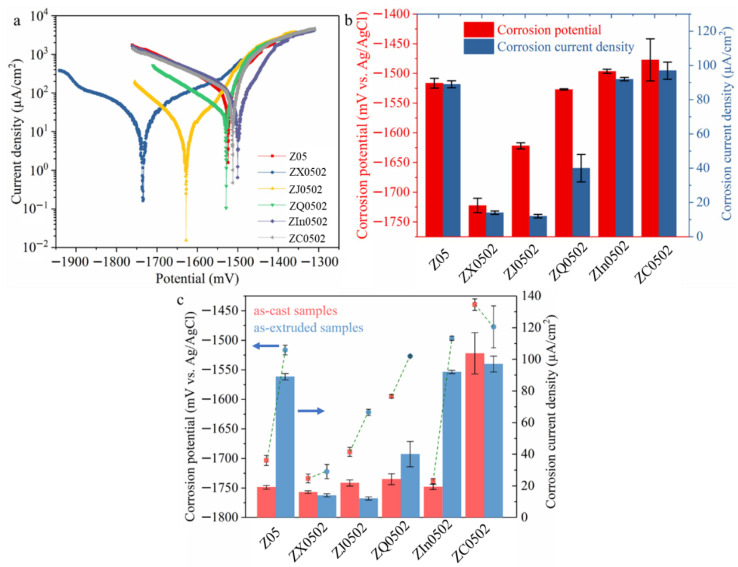
(**a**) PDP curves, (**b**) E_corr_ and i_corr_ values of the EX Mg-0.5Zn(-0.2X) alloys, (**c**) comparisons of E_corr_ and i_corr_ between the as-cast and EX counterparts. The E_corr_ and i_corr_ values of the as-cast samples are adapted from ref. [30].

The PDP curves (Figure 6) show that the E_corr_ values of Z0502-Ca (−1720 mV) and Z0502-Sr (−1620 mV) are the most and the second most negative, respectively, while those of Z05, Z0502-Ag, Z0502-In and Z0502-Cu are comparable (~−1500 mV). Moreover, Z0502-Ca and Z0502-Sr also possess the lowest i_corr_ values, followed by Z0502-Ag, while the values of Z05, Z0502-In and Z0502-Cu are similar (90–100 µA/cm^2^). Compared to the as-cast counterparts, the E_corr_ values of the EX alloys experience shifts to the positive direction, with the exception of Z0502-Ca and Z0502-Cu. In particular, the E_corr_ shifts in Z05 and Z0502-In reach as high as 200 mV. The i_corr_ values increase remarkably after hot processing, especially for Z05 and Z0502-In, where a four-fold raise can be observed.

**Figure 7:**
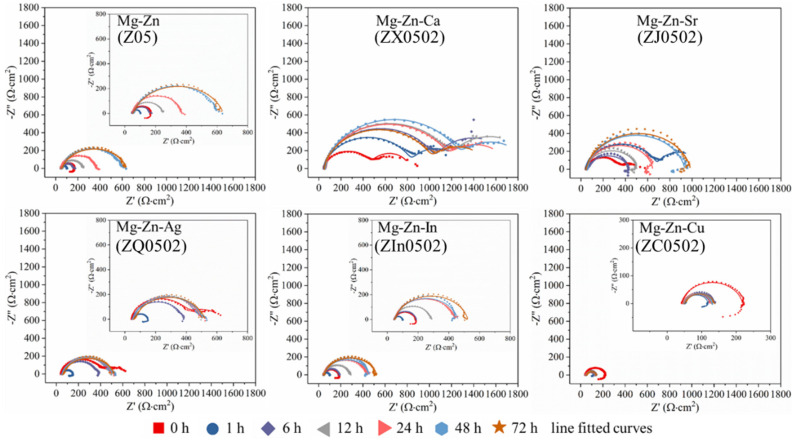
EIS Nyquist plots of the EX Mg-0.5Zn(-0.2X) alloys during 3 days of immersion.

**Figure 8:**
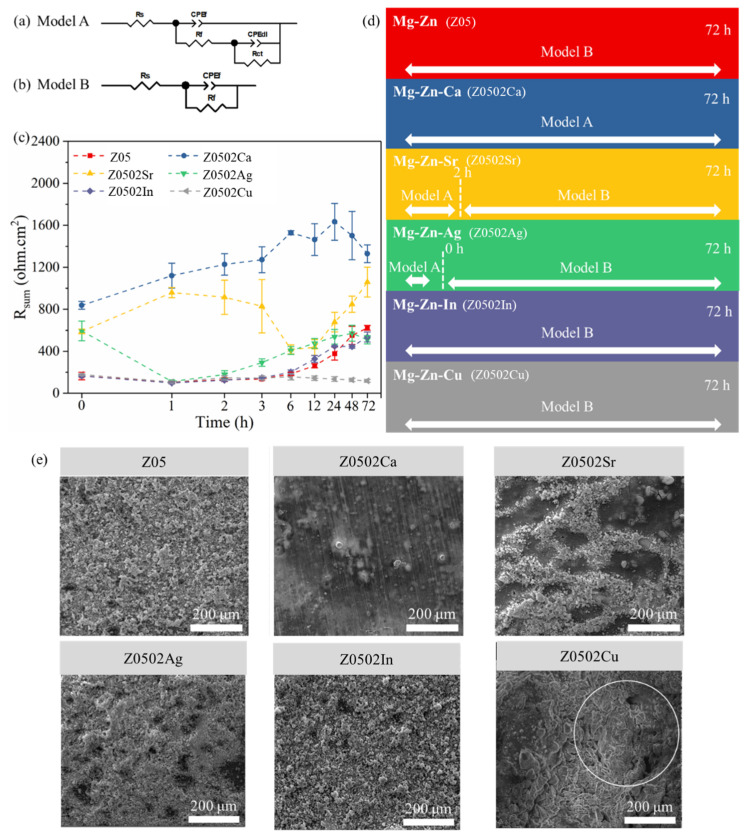
(**a**,**b**) Equivalent circuit models utilized to fit the EIS spectra of the EX Mg-0.5Zn(-0.2X) alloys; (**c**) change in the fitted R_sum_ during the 3 days test; (**d**) model selections at different immersion time intervals; (**e**) corresponding corrosion morphologies after 3 days.

The Nyquist plots of the EX Mg-0.5Zn(-0.2X) alloys are revealed in Figure 7 and the EIS data (capacitive components) is fitted with the equivalent circuit models in Figure 8. The general corrosion performances are evaluated by the sum of resistances, which is the sum of film resistance and charge transfer resistance (R_sum_ = R_f_ + R_ct_). Z05 and Z0502-In suffer severe corrosion attacks at the beginning of immersion with clear inductive loops in the low frequency ranges. The R_sum_ values decreasing to the minimum (~100 Ωcm^2^) at 1 h and then increasing steadily afterwards is possibly ascribed to the accumulation of the corrosion products. The steadily raising R_sum_ of Z0502-Ca implies the progressive build-up of corrosion products in the first 24 h (1459 Ωcm^2^), although it suffers slight decline at 72 h. The R_sum_ of Z0502-Sr increases within the first hour, followed by a decline until 6 h, possibly due to some localized corrosion from oxides film breakdown. The highest R_sum_ value for Z0502-Sr occurs after 72 h immersion (1058 Ωcm^2^). The R_sum_ of Z0502-Ag reaches its highest (595 Ωcm^2^) at the first 5 min of the measurement. Afterwards, R_sum_ decreases to the minimum (112 Ωcm^2^) at 1 h and then gradually increases again. R_sum_ maintains low (150 Ωcm^2^) during the entire immersion period of Z0502-Cu, indicating its persistently high corrosion rate. The low R_sum_ of Z05, Z0502-In and Z0502-Cu after 5 min hints that the corrosion kinetics are already strong at the interfaces in the initial stage of immersion.

The corrosion morphologies after 3 days immersion are shown in the insets in Figure 8d. The whole surfaces of Z05, Z0502-Ag and Z0502-In are corroded and covered with loose corrosion products. Z0502-Cu is the most severely corroded among all systems and is covered with thick corrosion products. Moreover, hundreds of pits of micron can be recognized (white circle). Filiform corrosion characteristics are observed in a partial area of Z0502-Sr. In contrast, Z0502-Ca is barely corroded, with only minor corrosion spots existing. The parallel patterns from grinding before corrosion can still be clearly seen.

**Figure 9:**
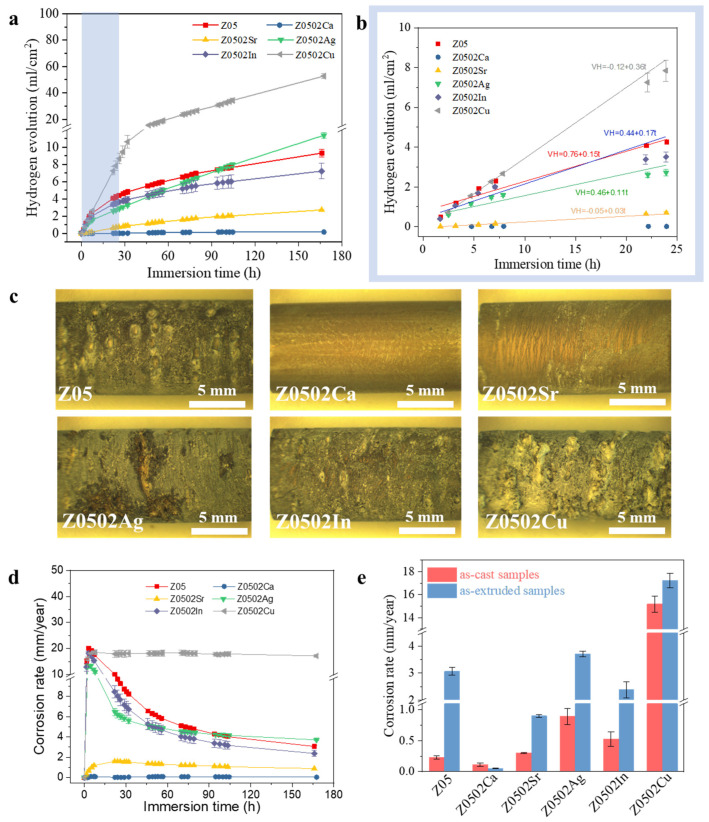
(**a**) Evolved hydrogen volumes of the EX Mg-0.5Zn(-0.2X) alloys during 7 days; (**b**) V(H_2_)-t dependencies of the alloys during the first 24 h; (**c**) corrosion morphologies of the samples after 7 days; (**d**) corrosion rates of the samples during 7 days of hydrogen evolution test; (**e**) corrosion rate comparisons of the as-cast and EX samples after 7 days immersion. The corrosion rates of the as-cast samples are adapted from previous work (ref. [30]).

A hydrogen evolution test was further conducted on the EX Mg-0.5Zn(-0.2X) alloys for 7 days to evaluate their corrosion performances (Figure 9). Only slight corrosion took place in EX Z0502-Ca, with the H_2_ generation efficiency being 0.16 mL/cm^2^ after 7 days immersion. The evolved H_2_ volumes from Z0502-Sr increase steadily to 2.71 mL/cm^2^ at 7 days. In contrast, H_2_ is generated vigorously in Z05, Z0502-Ag, Z0502-In and Z0502-Cu, especially at the early initial stage of immersion. The V(H_2_)-t exhibit approximately linear dependencies in the first 24 h, and the fitting equations are labeled correspondingly. The linear relationship is also reported elsewhere [45]. The slope represents the kinetics of the corrosion reaction. During the initial activation stage, corrosion at the surface/interface of Z0502Cu is the strongest, and that of Z0502, Z0502In and Z0502Ag are at a similarly high level. The final evolved H_2_ volumes from Z05, Z0502-Ag, Z0502-In and Z0502-Cu are 9.3, 11.4, 7.2 and 52.5 mL/cm^2^, respectively. The calculated corrosion rates of Z05, Z0502-Ca, Z0502-Sr, Z0502-Ag, Z0502-In and Z0502-Cu after 7 days are 3.1, 0.06, 0.9, 3.7, 2.4 and 17.2 mm/year, respectively. Slight corrosion occurs in EX Z0502-Ca, whereas the most severe corrosion takes place in Z0502-Cu. The EX Z05, Z0502-Ag and Z0502-In samples also suffer severe localized corrosion such as pitting and selective dissolution. Filiform-like corrosion is observed on the surface of the EX Z0502-Sr sample, with some places being preferentially corroded. Similarly to the cases in the as-cast states, Z0502-Ca possesses the highest corrosion resistance, whereas Z0502-Cu possesses the lowest corrosion resistance among the EX alloys. Specifically, the corrosion rate of Z0502-Ca derived from a hydrogen evolution test (0.05 mm/year) is even lower than that of the as-cast sample (0.12 mm/year). In contrast, the corrosion performances of Z05, Z0502-Ag and Z0502-In are notably deteriorated after hot processing.

## 4. Discussion

### 4.1. Microstructure

The possibility of defects pickup during the deformation process is reported [46]. However, as inferred from the similar element concentrations in the as-cast [30] and EX state (Table 2), the potential effect of contamination during solution annealing and extrusion on the corrosion performance can be excluded in this study.

Zn-containing intermetallics and segregations are generally dissolved into the matrix after annealing and extrusion for all Mg-Zn(-X) alloys, which is consistent with the literature [47,48]. Similarly to the as-cast Mg-Zn(-X) alloys [30], Si impurity still participates actively in the formation of intermetallics in the EX alloys. Only Mg_2_Si and Fe-Si precipitates are found in the extruded Z05, Z0502-In and Z0502-Ag. MgCaSi is the only intermetallic in the EX Z0502-Ca sample, which conforms to the strong bonding of Ca-Si [49]. The ΔH of α-Si_2_Sr (−42.7 kJ/mol) and β-Si_2_Sr (−40.3 kJ/mol) are lower compared to that of SiSr_2_ (−39.7 kJ/mol) [50]. Thus, the SiSr_2_(Zn) phase in as-cast Z0502-Sr might partially transform into Si_2_Sr phase during homogenization and extrusion. MgSrSi still remains after annealing and extrusion, revealing its high thermostability. Mg_2_Cu is the main precipitate in EX Z0502-Cu, while MgZnCu phases from its as-cast counterpart are not completely dissolved. The stability of the MgZnCu phase (withstanding the homogenization and extrusion processes) is also reported elsewhere [51,52]. In addition, Fe-containing precipitates are discovered in all extruded Mg-Zn(-X) samples. As the solubilities of Fe in Mg-Zn(-X) systems are limited at the heat treatment temperatures, Fe precipitations from α-Mg would take place [18].

**Figure 10:**
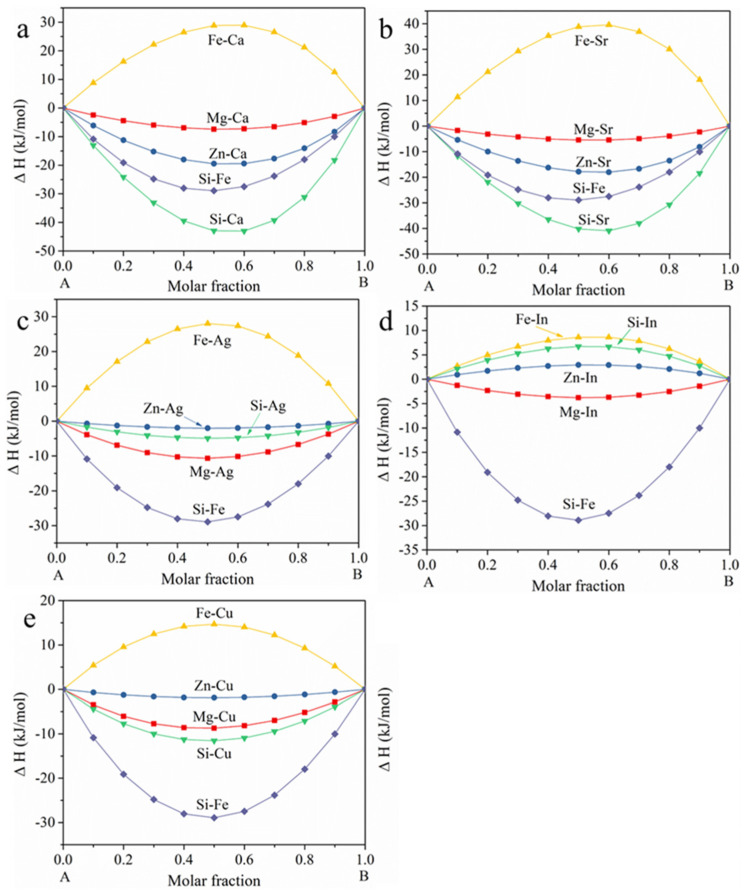
Thermodynamic calculation of the ΔH of A-B binary systems in (**a**) Z0502-Ca, (**b**) Z0502-Sr, (**c**) Z0502-Ag, (**d**) Z0502-In and (**e**) Z0502-Cu. Those of Mg-Zn, Mg-Si, Mg-Fe, Zn-Si and Zn-Fe are already calculated in [18].

Thermodynamic calculations of the ΔH (enthalpy of formation) of A-B binary systems in Mg-Zn-X alloys are collectively presented in Figure 10. Si could form compounds with Mg and alloying element X with the exceptions of Zn and In, while Fe impurity interacts preferentially only with Si, with the ΔH values of Fe-Mg, Fe-Zn and Fe-X being either positive or near 0. The alloying systems tend to transfer to the lower ΔH values aiming for higher stabilities. During solution annealing, the elements have time to diffuse and homogenize thoroughly.

The ΔH value of Si-Fe is the lowest in the Z05 [18], Z0502-Ag and Z0502-In systems; thusly, Fe precipitation takes place on the Mg_2_Si phase during hot processing, as Si and Fe have strong bonding strengths. The ΔH of Si-Fe is also the lowest in the Z0502-Cu system, and that of Si-Cu comes the second most negative, leading to Fe precipitation in the MgSiCu phase. In contrast, the ΔH sequences in the Z0502-Ca and Z0502-Sr systems differ. The ΔH of Si-Ca and Si-Sr are more negative than that of Si-Fe, leading to the formations of MgCaSi(Fe), Si_2_Sr(Fe) and MgSrSi(Fe) precipitates in the respective systems (Figure 5b,c). The potential intermetallics in the as-cast and EX Mg-0.5Zn(-0.2X) alloys are summarized in Table 4. The distribution of Fe impurity is also shown.

The texture intensity of Z05 is reduced by adding Ca and Sr (Figure 3), especially in the case of Ca. The thermostable MgCaSi phase can facilitate recrystallization during deformation due to the accumulating dislocations in the grains (PSN), and the recrystallized grains exhibit more random textures [53]. In addition, Ca (197.4 pm) has a bigger atomic radius than Mg (159.9 pm) [54]; the dissolution of Ca during hot processing can decrease the stacking fault energy [55] and axial ratio (c/a) [56], resulting in the generation of non-basal slip and the weakening of texture characteristics. As the solubility of Sr is much lower than that of Ca [57], the texture intensity of Z0502-Sr is higher than that of Z0502-Ca. All the other extruded Mg-0.5Zn(-0.2X) samples exhibit typical fiber textures.

### 4.2. Corrosion Performance

Theoretically, an alloy with few intermetallics has limited galvanic corrosion. However, the presence of Fe-Si precipitates significantly impairs the corrosion resistances of EX Z05, Z0502-Ag and Z0502-In compared to their as-cast states. As the potential difference (ΔE) of Mg_2_Si phase with respect to the matrix is low [58,59], the incorporation of Fe [60] (675 mV higher than matrix) or Fe_3_Si [61] (239 mV higher than matrix) would promote the overall ΔE value significantly and generate strong galvanic corrosion kinetics. The formed oxide layers of these alloys are not protective, as inferred from the relatively low R_f_ values (Figure 7). As no passivity exists, high grain boundary density will accelerate the corrosion rate, as the grain boundaries serve as the metallographic defects and are prone to corrosion [62,63,64].

The diminishment of intermetallics and Zn segregations after hot processing leads to a better corrosion resistance of Z0502-Ca. The sample undergoes “passivation” with the corrosion rate being 0.06 mm/year after 7 days of immersion (Figure 9). In this case, the grain boundary will facilitate the passivation process and build compact corrosion layers, thereby reducing the corrosion rate [62,63,64,65,66]. Although Fe precipitation also takes place during hot processing, the Fe-Si precipitates are encapsulated by MgCaSi in the EX sample, which is less damaging to the corrosion resistance. The influence of Fe or Fe_3_Si included in MgCaSi is not as great as that incorporated in Mg_2_Si, as MgCaSi has much higher ΔE [58] than Mg_2_Si. Additionally, the dissolution rate of the (0001) plane is much lower than those of the $(10\bar{1}0)$ and $(11\bar{2}0)$ planes, because of its higher in-plane atomic density and lower surface energy [67,68]. Lower fiber texture intensity means a higher possibility of the (0001) basal plane lying in parallel to the sample surface. Therefore, the weakened fiber texture intensity further contributes to the higher corrosion resistance of Z0502-Ca [69].

By virtue of the strong bonding of Sr-Si, the MgSrSi and Si_2_Sr phases withstand hot processing and some “stringer-like” precipitates occur. The long distances among the clusters contribute to a relatively less continuous corrosion layer in the EX sample [70,71]. Compared to Z05, Z0502-Ag and Z0502-In, the much better corrosion performances of Z0502-Sr might also be related to the impurity distributions. There are Fe precipitates in the MgSrSi and Si_2_Sr particles during heat treatment. Although the ΔE of these particles are still unavailable in the literature, they are expected to be higher than that of Mg_2_Si (similar to the case of MgCaSi), exerting a milder effect on the corrosion property. Additionally, the slightly weakened texture also helps to improve the corrosion resistance.

The corrosion resistances of the cast and extruded Z0502-Cu are comparably low. Most MgZnCu phases change to Mg_2_Cu phases after annealing and extrusion, with the overall area fractions of the precipitates being similar. The much nobler corrosion potential of Mg_2_Cu (−1 V_SCE_) compared to pure Mg (−1.68 V_SCE_) [72] indicates accelerated galvanic corrosion would take place.

### 4.3. Perspective: Design Strategy of Micro-Alloyed Mg Systems

As discussed above, the intermetallics, textures and impurities are closely related to the corrosion performances of Mg alloys. As for the micro-alloyed Mg systems, the influences of intermetallics and textures are limited, leaving the distribution of impurities as the most critical. The maximal solubility of Fe and Si in Mg is only 10 and 30 ppm, respectively [54,73]. The Si/Fe-ratio tolerance limits in the common high purity Mg alloys are still not clear. Even traces of Si could significantly decrease the tolerance limit of Fe in Mg and accelerate the corrosion rate [25,39,74]. Corrosion problems associated with Fe and Si inclusions remain to be solved, as the availability of extra-high purity Mg is quite limited.

**Figure 11:**
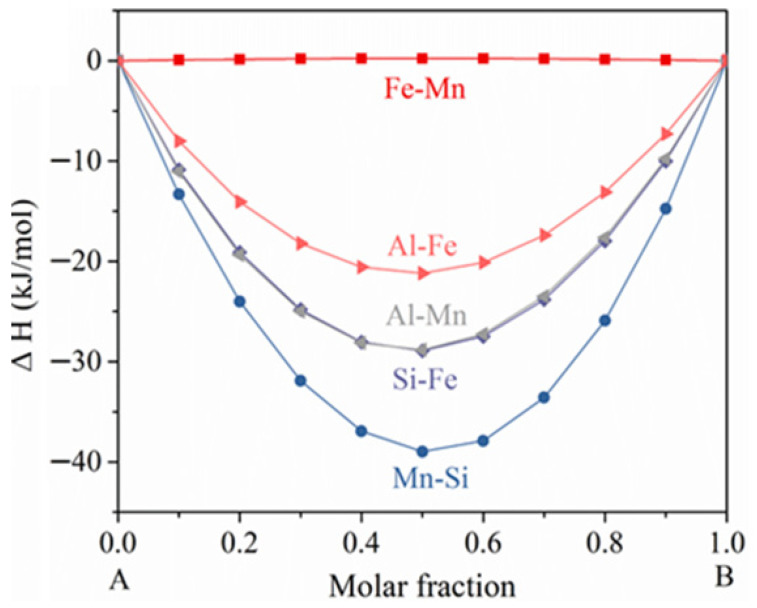
Thermodynamic calculation of the formation enthalpies of A-B binary systems in Al-Mn-Si-Fe.

For the alloys with a certain amount of Fe and limited intermetallics, Fe precipitation during heat treatment cannot be ignored. To suppress the incurred negative effects, some restriction should be met (with Mg-X-Fe as an example). Firstly, the formation of enthalpy should be in the following order: ΔH (Mg-X) < ΔH (Fe-X) < η (η is a negative value which ensures the strong bonding of Fe-X), so that the supersaturated Fe can precipitate in Mg/X intermetallics during heat treatment. However, this is not the full story. Mg/X must also have an appreciable ΔE so that the corrosion rate of the Mg-X alloy will not be significantly promoted. When it comes to ternary or even more complex systems (e.g., Mg-X-Y-Fe), Mg does not necessarily participate in the intermetallic formation and thus the restrictions can be adjusted, as ΔH (X-Y) < ΔH (Fe-X) < η and ΔE (X/Y) is not low. It is widely recognized that the addition of Mn is propitious to the corrosion performance of Mg-Al alloy. Hence, with the Al/Mn containing Mg alloy as an example, it is found that ΔH (Al-Mn) < ΔH (Al-Fe) << 0 (Figure 11) and Al_8_Mn_5_ has a high ΔE value (340 mV [75], 385 mV [76], 487 mV [59]); thus, the Al_8_Mn_5_ phase can encapsulate Fe during hot processing [24] and will not accelerate the corrosion rate significantly. As for the Al-free alloys in this study, it is expected that the additions of Mn would also reduce the negative effects of Fe precipitation due to ΔH (Mn-Si) < ΔH (Si-Fe) << 0 (Figure 11) and the notable ΔE of Mn_5_Si_3_ (428 ± 148 mV) [58]. Further experimental studies are still needed to validate the aforementioned assumption. It is recommended to set up the database regarding the ΔH and ΔE of the precipitates and impurities. Without conducting experiments, it might be predictable to see whether hot processing has major negative impacts on the corrosion performances of the alloys via methods such as artificial neural networks [77]. It is also important to mention that the Miedema model is a semi-empirical approach and certain deviations might exist for the values calculated in this study and those from other theories. For example, Wu et al. [49] used density functional theory to calculate the ΔH of MgCaSi, and the value was −0.4766 eV/atom. As 1 eV/atom ≈ 96.485 kJ/mol, the formation enthalpy is determined as −0.4766 × 96.485 ≈ −46 kJ/mol, which is close to the value (−43 kJ/mol) in this work. Nevertheless, the focus of this paper is not to quantitatively determine the precise value of formation enthalpy, but to unveil the ordering of the bonding tendency of the alloying elements and impurities from a thermodynamic method.

## 5. Conclusions

This study investigates the interactions between intermetallics and impurities during hot processing in micro-alloyed Mg systems, and the corresponding influence on corrosion performance. The main conclusions are as follows:

(1) After hot processing, the phenomenon of Fe precipitation can be seen in micro-alloyed Mg systems.
(2) The corrosion performances of as-extruded Z05, Z0502-Ag and Z0502-In deteriorate distinctly compared to the as-cast states due to the redistribution of Fe precipitates.
(3) The influence of Fe precipitation during hot processing is not that severe for Z0502-Ca and Z0502-Sr, mainly resulting from the lower ΔH of Ca-Si and Sr-Si compared to Fe-Si, and the appreciable ΔE of the MgCaSi and MgSrSi phases.
(4) Regarding the corrosion performance of micro-alloyed Mg alloy, ΔH and ΔE of the precipitates are the key factors during hot processing. A corresponding database would be desired in the perspective of material design.

## Figures and Tables

**Table 1:** Biocompatiblity and solubility of the selected alloying element [30].

Element	Ca	Sr	Ag	Cu	In
Biocompatibility	Beneficial for bone regeneration and suppressing bone resorption		The released Ag^+^ and Cu^2+^ have antibacterial effect		Commonly added in alloys for dental materials
Maximal solubility in Mg (wt.%)	1.34	0.11	15	0.0034	53

**Table 2:** Main concentrations of the extruded Mg-0.5Zn(-0.2X) alloys identified using ICP-OES.

Element	Z05	ZX0502	ZJ0502	ZQ0502	ZIn0502	ZC0502
Zn (wt.%)	0.49 ± 0.02	0.51 ± 0.02	0.48 ± 0.02	0.50 ± 0.02	0.49 ± 0.02	0.47 ± 0.02
X (wt.%)	-	0.18 ± 0.02	0.19 ± 0.02	0.19 ± 0.02	0.19 ± 0.02	0.20 ± 0.01
Mn (ppm)	169 ± 5	200 ± 5	186 ± 5	168 ± 5	171 ± 5	174 ± 5
Si (ppm)	67 ± 4	137 ± 4	154 ± 4	140 ± 4	57 ± 4	59 ± 4
Al (ppm)	32 ± 5	52 ± 5	130 ± 5	146 ± 5	81 ± 5	55 ± 5
Fe (ppm)	13 ± 6	22 ± 6	13 ± 6	13 ± 6	12 ± 6	7 ± 6
Cu (ppm)	<3	<3	<3	<3	<3	-
Ni (ppm)	<3	<3	<3	<3	<3	<3
Be (ppm)	<3	<3	<3	<3	<3	<3

**Table 3:** Influence of extrusion speeds on the grain sizes of EX Mg-0.5Zn(-0.2X) alloys.

	Speed	0.6 mm/s	2.2 mm/s	4.4 mm/s
Alloy				
Z05		26 ± 1 µm	30 ± 1 µm	31 ± 1 µm
Z0502Ca		7 ± 1 µm	20 ± 1 µm	30 ± 2 µm
Z0502Sr		23 ± 2 µm	26 ± 0 µm	31 ± 1 µm
Zn0502Ag		24 ± 0 µm	26 ± 0 µm	29 ± 1 µm
Z0502In		24 ± 0 µm	27 ± 2 µm	30 ± 2 µm
Z0502Cu		22 ± 1 µm	32 ± 1 µm	37 ± 1 µm

**Table 4:** The potential intermetallics in the as-cast and EX Mg-0.5Zn(-0.2X) alloys.

Alloy	As-Cast	EX	
	Intermetallics	Intermetallics	Form of Fe Impurity
Z05	Mg_2_Si, MgZn	Mg_2_Si	Fe-Si
Z0502-Ca	Mg_2_Ca, Ca_2_Mg_6_Zn_3_, MgCaSi	MgCaSi	MgCaSi(Fe)
Z0502-Sr	MgSrSi(Zn), SiSr_2_(Zn)	MgSrSi, Si_2_Sr	MgSrSi(Fe), Si_2_Sr(Fe)
Z0502-Ag	Mg_2_Si, Zn_5_Ag	Mg_2_Si	Fe-Si
Z0502-In	Mg_2_Si, MgZn	Mg_2_Si	Fe-Si
Z0502-Cu	Mg_2_Si, MgZnCu	Mg_2_Cu, Mg_2_Si, MgSiCu, MgZnCu	Fe-Si

## Data Availability

The original contributions presented in this study are included in the article. Further inquiries can be directed to the corresponding author.
